# Measuring IGF-1 and IGFBP-3 Profiles in Women Seeking Assisted Reproduction; Relationship to Clinical Parameters (Study 1)

**DOI:** 10.3390/jpm10030122

**Published:** 2020-09-11

**Authors:** John L. Yovich, Syeda Zaidi, Minh D. K. Nguyen, Peter M. Hinchliffe

**Affiliations:** 1 PIVET Medical Centre, Perth 6007, WA, Australia; nureenazaidi@yahoo.com (S.Z.); mdknguyen@pivet.com.au (M.D.K.N.); apwin@pivet.com.au (P.M.H.); 2 Department of Pharmacy and Biomedical Sciences, Curtin University, Perth 6845, WA, Australia

**Keywords:** body mass index (BMI), stature (height), age groups, IGF-1 profile, IGFBP-3/IGF-1 ratio (IGF Ratio), assisted reproductive technology (ART), in vitro fertilization (IVF), growth hormone (GH)

## Abstract

This study examines the IGF serum profile (IGF-1, IGFBP-3 and the IGF Ratio) from 1633 women who undertook an Assessment Cycle prior to any treatment by assisted reproduction. The idea is to progressively study the IGF profile with a view to identify those women who may be classified as having adult growth hormone deficiency (AGHD) and who may benefit from specific dynamic endocrinological testing to identify a potential benefit from growth hormone adjuvant treatment. This first study evaluates the IGF profile on clinical parameters, namely age, body mass index (BMI) and stature. The study shows a significant linear reduction in IGF-1 levels across the four age groups (<35 years, 35–39 years, 40–44 years and ≥45 years; *p* < 0.001). However, there was no variation in IGFBP-3 levels but the IGF Ratio showed a progressive linear elevation with advancing age (*p* < 0.001). With respect to both BMI and stature, none of the IGF profile parameters showed any variation. We conclude that further studies are warranted to examine the notion of underlying AGHD in the causation of the well-known feature of age-related poor prognosis in assisted reproduction.

## 1. Introduction

The assisted reproductive technology (ART) program at the PIVET Medical Centre is one of the founding pioneer facilities and has been operational since 1980 [1,2]. Specific aspects of interest in the management of infertility patients is the preliminary undertaking of an Assessment Cycle prior to initiating any treatment regimen [3], and thereafter, those patients directed into an in-vitro fertilization (IVF) program are managed according to a dedicated Algorithm, which specifies the ovarian stimulation schedule and dosage, the type of ovulatory Trigger and dosage and the luteal phase management protocol. The latter is designed to optimize mid-luteal hormone levels for embryo implantation. With a committed single embryo transfer policy and the aim to cryopreserve, by vitrification, residual high-quality blastocyst embryos, PIVET measures its success rate by a live birth productivity index, also sometimes referred as one type of cumulative livebirth rate [4]. This means a calculation identifying those pregnancies accumulated from both fresh and frozen embryo transfers and which progress beyond the gestational stage of 20 weeks, as a rate of each single treatment cycle with ovarian stimulation initiated. The aim of the PIVET Algorithm is to collect 10 ± 2 oocytes, thereby optimizing the chance for a livebirth outcome whilst minimizing the risk of ovarian hyper-stimulation [5]. However, whilst this objective is consistently achieved in the PIVET IVF program, the prognosis for livebirth is variable being mainly, albeit not entirely, an age-related phenomenon. We have projected the hypothesis that growth hormone (GH) deficiency may underlie this variability in IVF prognosis [6]; hence, we have undertaken sequential studies attempting to support this. This first study, presented here, examines the IGF-1 and IGFBP3 profiles in women attending with infertility, and prior to any fertility treatment. It is established that IGF profiles provide the foundation for the diagnosis of GH deficiency in childhood [7]; hence, we have undertaken similar screening for the women presenting for ART. Our subsequent studies will report IGF-1 responses to GH treatment, and thereafter, the pregnancy productivity according to IGF-1 levels with/and without GH adjuvant treatment.

## 2. Materials and Methods

Women attending PIVET for infertility management have height and weight estimations for body mass index (BMI) calculation at their primary consultation along with the collection of demographic and historical medical information. All patients and their partners undergo physical medical examinations, and they are encouraged to undergo a preliminary Assessment Cycle (AC) during which several tests are undertaken relevant to their infertility problem and its potential management [3]. A morning blood sample is collected around day-5 of an unstimulated menstrual cycle and spun down immediately for the estimation of Insulin-like Growth Factor-1 (IGF-1) as well as its main binding protein Insulin-like Growth Factor Binding Protein-3 (IGFBP-3) in the serum, being the main one of 6 binding proteins described. Although overnight fasting would be ideal, patients are advised to have no more than a light breakfast, such as “tea and toast” on the morning of the test. In addition, other hormonal tests are conducted on the serum sample including anti-Mullerian hormone (AMH), and the woman has a trans-vaginal ultrasound (TVUS) procedure which includes an estimation of the antral follicle count (AFC) in her ovaries. The AC includes ovarian tracking by pre-ovulatory TVUS and serum hormonal measurements of both estradiol and progesterone to define the pre-ovulatory phase for a post-coital test. The woman is thereafter reviewed around Day-21 when specific fertility management is planned.

The women selected for this study are drawn from PIVET’s database which lists IVF cases commencing from 1981. However, the period selected is from 1 January 2011 to 31 December 2019. This 9-year period embraces consistency within the laboratory and clinical program focusing on blastocyst culture (~90%), cryopreservation (~65%), exclusively by vitrification applying the Cryotop^®^ technique, and a high commitment to single embryo transfers, currently conducted on more than 95% of fresh and frozen cycles. The preliminary ACs are performed on more than 60% of cases with IGF-1, IGFBP3, AMH and AFC as well as BMI calculations tabulated within the Filemaker^®^ database program. Both AMH and AFC Groupings were available for all these women.

### 2.1. IGF-1 and IGFBP-3 Assays

The IGF-1 and IGFBP-3 immuno-assays were conducted on separate platforms. Venous blood is drawn into EDTA-containing tubes for plasma separation. The tubes are immediately chilled. The IGF-1 assay is a one-step chemiluminescent immunoassay applying a sandwich technique using a monoclonal antibody. The LIASON^®^ platform adopts a “flash” chemiluminescence technology with a para-magnetic microparticle solid phase (DiaSorin, Saluggia (VC) Centralino, Italy). The IGFBP-3 is measured on a solid-phase, enzyme-labelled chemiluminescent immunometric assay (IMMUNOLITE 2000; Siemens Healthcare GmbH, Erlangen, Germany). These assays were undertaken courtesy of Clinipath Pathology, Perth, Western Australia 6017.

### 2.2. IGFBP-3/IGF-1 (IGF Ratio)

Applying the formula: IGFBP-3/IGF-1 provides a ratio (IGF Ratio), which has clinical implication for the diagnosis of GH disorder where the optimal range should be 1.6 to 4.5; for our practical purpose, a Ratio > 5.0 implies that investigation for GH deficiency should be considered. In Australia, GH treatment is subsidized under Medicare if dynamic testing by an Endocrinologist demonstrates adult Growth Hormone deficiency (AGHD).

### 2.3. Statistical Analysis

Data extractions from the Filemaker^®^ database were assembled in Microsoft Excel spreadsheets and sorted according to the relevant tests. Thereafter, the sorted data were placed in the application Past 4.03 (developed by Øyvind Hammer) [8] for statistical data analysis. This application also generated the Tables comprising the statistical summaries, finally placed in Microsoft Word for clearer display. Having demonstrated that the data comprising the IGF profile (IGF-1, IGFBP-3 and IGF Ratio) are all distributed in a Normal fashion, the relationship among the means was examined by one-way ANOVA for overall comparison. Both Mann–Whitney and Tukey’s pair-wise plots compared the individual means which ranged from three (in percentile studies of stature) to eight (in BMI comparisons) for various analyses. The Kruskal–Wallis test was applied to examine equality between sample medians. Probability values *p* < 0.05 were considered significant for any test. The Past 4.03 application also generated the figures which were then upgraded in the xDiagram^®^ 5.4 application (developed by Vu Tien Thinh) enabling optimal display for this publication.

### 2.4. Ethical Considerations

PIVET is accredited with both the self-regulatory National Australian Reproductive Technology Committee (RTAC) as well as the Reproductive Technology Council (RTC) of Western Australia. Reporting of the data was approved under Curtin University Ethics Committee approval NO. RD_25–10 general approval for retrospective data analysis in 2010, updated in 2015.

## 3. Results

**Figure 1:**
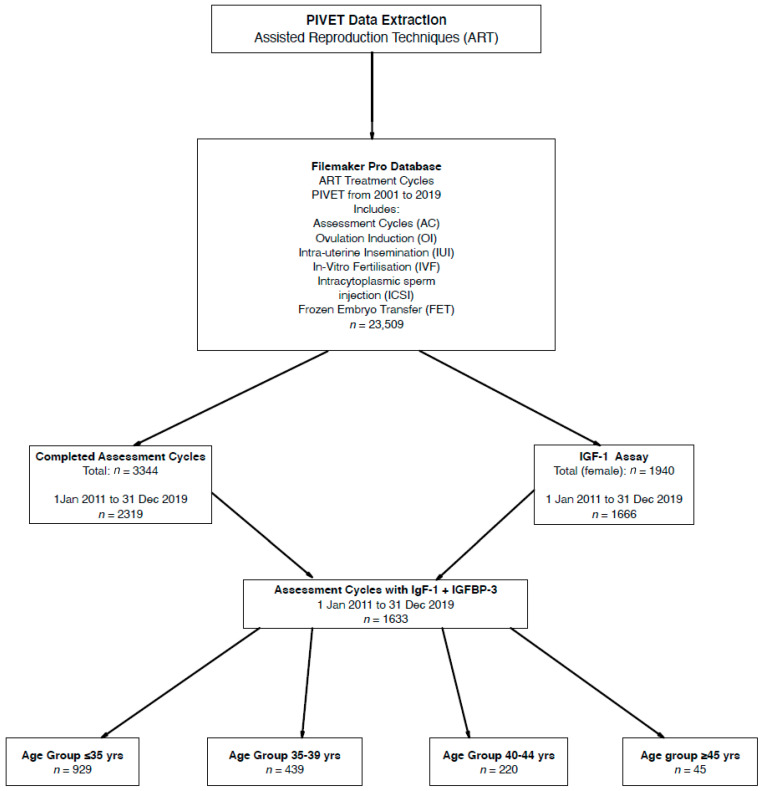
Flow diagram showing derivation of 1633 women who had IGF-1 and IGFBP-3 in early follicular phase of an Assessment Cycle undertaken prior to any definitive treatment.

Here we are reporting in SI units (Système Internationale; International System), and these are applied in our clinical practice. The conversion of SI units nmol/L to conventional units (ng/mL) is 7.65; hence, 25 nmol/L can be read as 191 ng/mL. This conversion factor applies for both IGF-1 and IGFBP-3. As can be seen from the Flowchart (Figure 1), 1633 women had IGF profiles (IGF-1, IGFBP-3 and IGF Ratio) measurements performed within an AC and both Age and BMI calculations were available for all these women at the time of the test. Both height and weight were measured for all these women, enabling the BMI calculation, and height was specifically examined in the study related to stature.

### 3.1. IGF Profile

**Figure 2:**
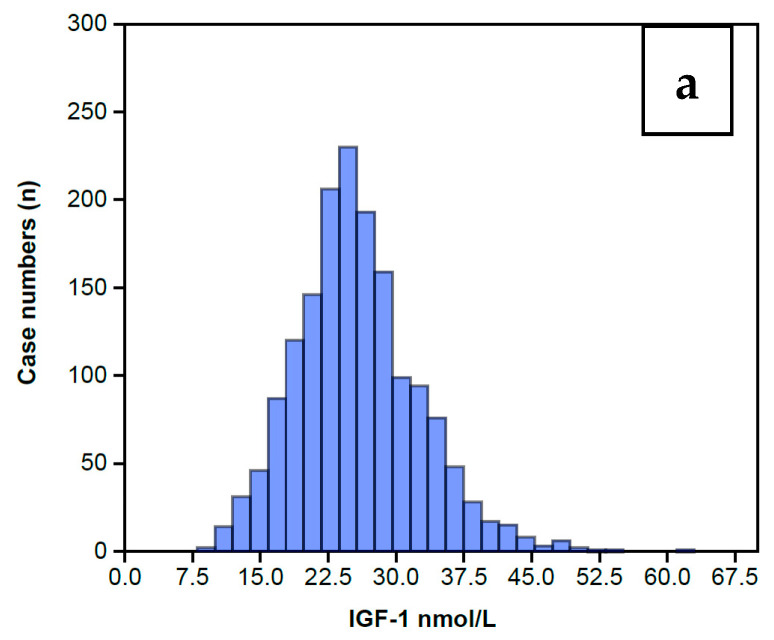

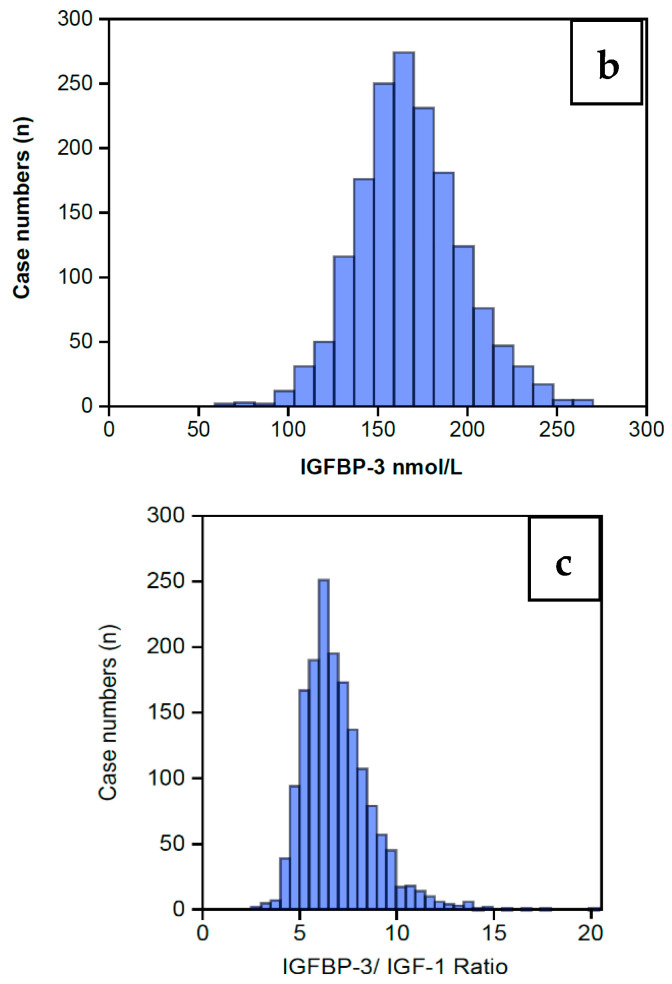
Histograms showing the overall IGF-1 profile for those 1633 women who completed the IGF-1 assay (**a**) along with the IGFBP-3 assay (**b**) on the same serum sample. The IGFBP-3/IGF-1 ratio is calculated from these levels (**c**). It can be seen that all the histograms display a Normal distribution, albeit with some minor skew to the right, maximally pronounced in the Ratio.

The distribution of serum IGF-1 levels for the 1633 women completing an AC prior to any treatment is shown in Figure 2 as a Normal distribution centered around a Median level of 25 nmol/L (Figure 2a) and a mean of 25.5 nmol/L with a standard error of 0.17. The full range of data is summarized in Table 1, showing that IGF-1 levels extend from a low of 8 nmol/L to a high of 61 nmol/L, and the inter-quartile range is 21 nmol/L to 29 nmol/L. The wider range embracing 2 standard deviations covers 12 nmol/L to 49 nmol/L. Table 1 also includes the IGFBP-3 data, which also displays a Normal distribution for this same group of 1633 women, shown in Figure 2b. The preceding data sets enabled calculation of the IGFBP-3/IGF-1 ratios (IGF Ratio), also depicted in Table 1. The IGF Ratio also displays a Normal distribution (Figure 2c), albeit with a minor skew to the right, ranging from a minimum 2.5 to a maximum 20.4, with a median level of 6.6. The inter-quartile range is from 5.7 to 7.8. The wider range embracing 2 standard deviations centers around the mean ratio of 6.9 with a tight standard error of 0.04 and covers a low ratio of 3.3 to a high ratio of 10.5.

### 3.2. IGF Profile vs. Age

**Figure 3:**
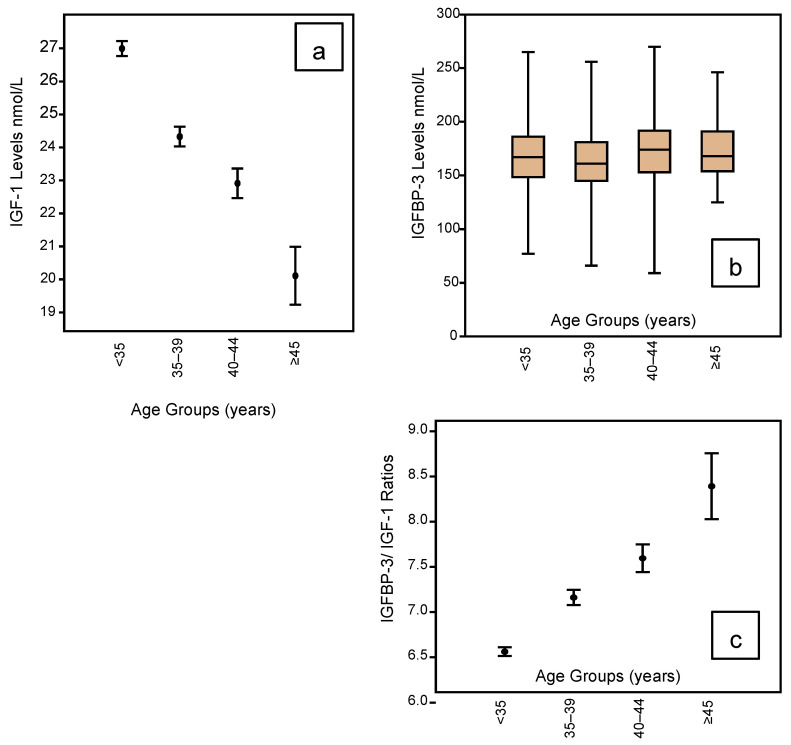
Mean and whisker diagrams showing the IGF-1 profile according to the age groupings of the 1633 women who completed an Assessment Cycle prior to any ART treatments. There is a linear reduction in the IGF-1 mean levels with advancing age (mean and whisker plot (**a**); *p* < 0.001), but there is no significant change in IGFBP-3 levels across the age ranges (box and whisker plot (**b**)). This translates to a significant rise in the IGFBP-3/IGF-1 ratio with advancing age (mean and whisker (**c**); *p* < 0.001).

The IGF profile according to age groupings is shown in Figure 3 embracing IGF-1 (Figure 3a); IGFBP-3 (Figure 3b); and the IGF Ratio (Figure 3c). It can be seen that IGF-1 levels are lowest in the <35-year age group, falling progressively and significantly with rising age. The mean levels fall from 27.0 in the youngest group to 20.1 for those women who have reached 45 years of age (Table 2). However, there is no such change in the IGFBP-3 profile across the age groups (Table 2), but this means the IGF Ratio rises progressively and significantly with rising age. Table 2 shows the mean IGF Ratio rising from 6.56 for the youngest to 8.39 for the oldest group.

### 3.3. IGF Profile vs. BMI

**Figure 4:**
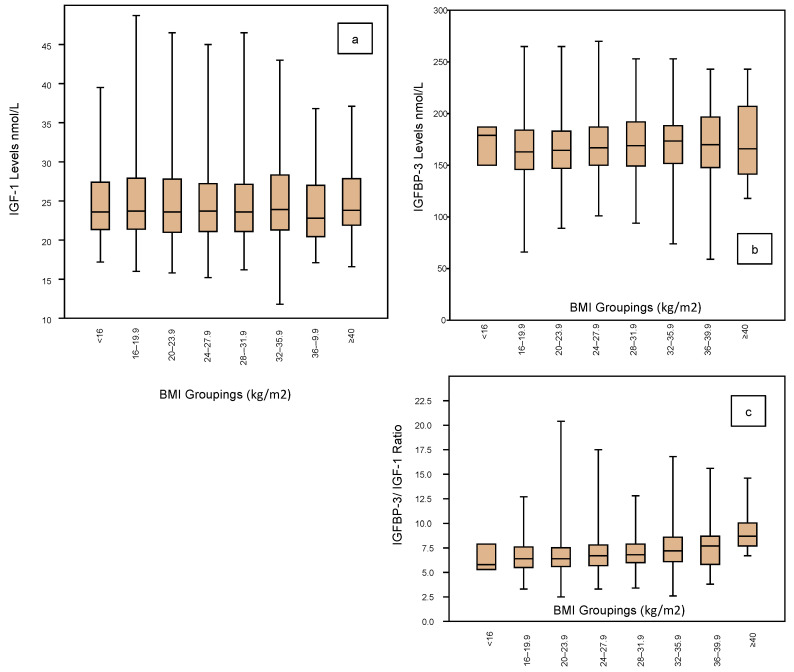
Shows the IGF-1 profile according to the BMI ratings (grouped by 4 units) of the 1633 women who completed an Assessment Cycle prior to any ART treatments. There is no significant variation in the IGF-1 mean levels across the BMI spectrum from <16 (comprising 3 cases only) to >40 (comprising 17 cases only) (**a**). Neither is there any significant change in IGFBP-3 levels across the BMI ranges (box plots; (**b**)). These unchanged mean levels therefore translate to a stable IGFBP-3/IGF-1 ratio across the BMI spectrum (**c**).

The IGF profile according to BMI groupings is shown in Figure 4 embracing IGF-1 (Figure 4a), IGFBP-3 (Figure 4b) and the IGF ratio (Figure 4c). The BMI groupings are categorized for every 4 kg, ranging from <16 kg/m^2^ to ≥40 kg/m^2^, but there are very few cases in the two extreme groups. There are no significant differences in IGF-1 levels across the groups, the mean being around 24 nmol/L (Table 3). Similarly, there are no significant differences in the IGFBP-3 levels across the groups, the mean being in the range of 168 ± 2 nmol/L (Table 3). Similarly, IGF Ratios show no significant differences across the BMI groups, being around 6.5 (Table 3).

### 3.4. IGF Profile vs. Stature

**Figure 5:**
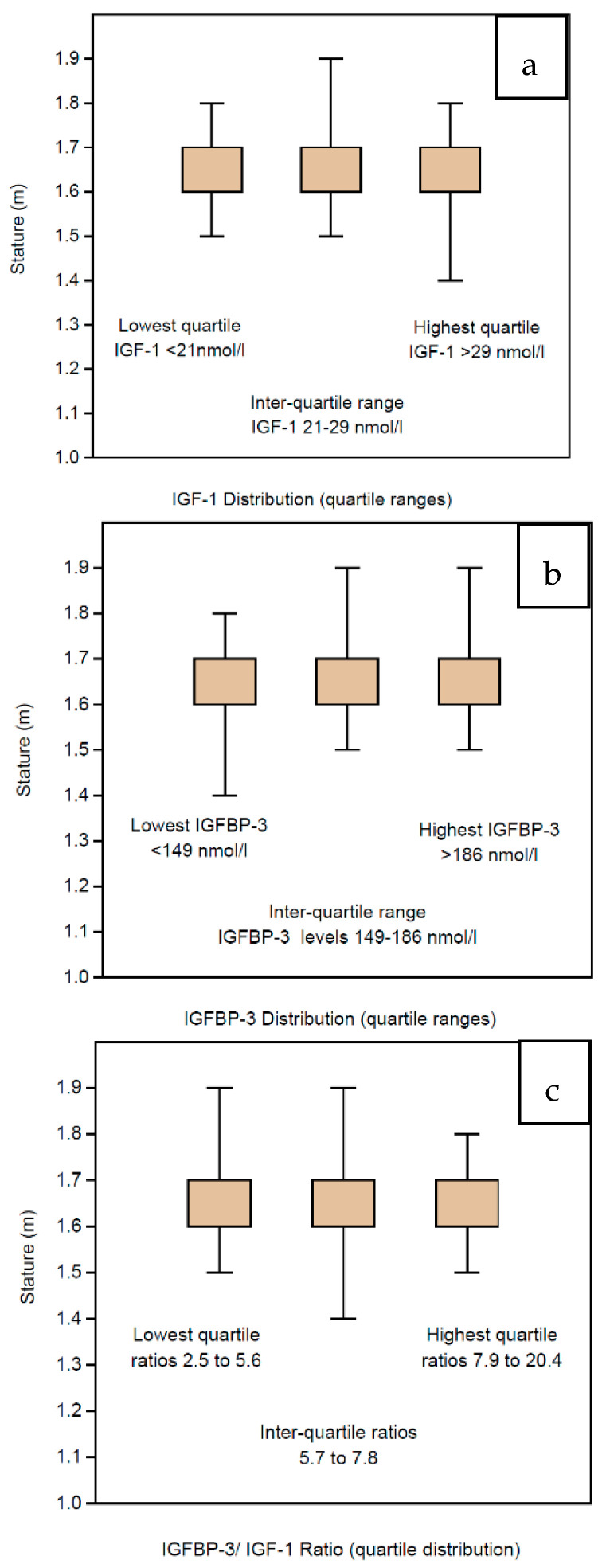
Box and whisker figures displaying the quartile distribution of IGF profiles for the 1633 women according to their stature measured in meters. There are no significant differences in stature across the quartiles for IGF-1 (**a**), IGFBP-3 (**b**) or the IGF Ratio (**c**).

Although the IGF profiles are stable across all the BMI groups, we have also sub-analyzed the profile according to the height of the women (which is already embedded in the formula for BMI estimation). In children, the main determinant for GH deficiency is short stature and suboptimal growth is clinically assessed by serial height measurements. The stature among the women in our study was assessed by measurements of heights, which have been categorized per 10 cm groupings ranging from 1.4 m to 2.0 m. The mean IGF-1 levels did not differ significantly across the height categories, ranging from 24.1 to 25.2 across the relevant range (Table 4). The statistical analysis excludes the highest range (1.9–2.0 m) comprising only 2 women, interestingly, with a lower mean of 23.0. In particular, the women with the shortest stature (<1.6 m) had mean IGF-1 levels >24.0, which were not lower than IGF-1 levels of the taller women (>1.7 m). There were 10 women whose IGF-1 levels were in the lowest region (under 2 standard deviations, being ≤11 nmol/L; they were also presented as clinically normal, with stature ranging from 1.5 to 1.7 m. Neither did the IGFBP-3 levels vary, the women having mean levels ranging from 163.3 nmol/L to 169.8 nmol/L in the height ranges from 1.5 to 1.8 m (Table 4). Of interest, the shortest women had the lowest mean level of IGFBP-3 at 153.1 nmol/L, and the tallest had the highest mean level of IGFBP-3 at 197.0 nmol/L, but these levels had no statistical relevance. IGF Ratios were also stable with mean levels ranging from 6.8 to 7.1 across the relevant, populated range (Table 4). Because there are very few cases in the two extreme groups, the data have also been presented in centiles—IGF-1, IGFBP-3 and IGF Ratio in Table 4. Consequently, the IGF profile with respect to stature among the women in our study is presented in quartile ranges, namely, IGF-1 in Figure 5a, IGFBP-3 in Figure 5b and IGF Ratio in Figure 5c.

## 4. Discussion

This study is the first of several from PIVET Medical Centre examining the relevance of testing the IGF profile (IGF-1, IGFBP-3 and IGF Ratio) of women attending for assisted reproduction. PIVET is a pioneer IVF facility and has published several studies since 2010 exploring the clinical use of GH as an adjuvant to improve IVF outcomes for women categorized as poor prognosis (6). Recently, our studies have progressed into an evaluation of the GH-IGF signaling process, demonstrating a convergence with gonadotrophin signaling [9,10]. In order to determine which women may benefit from GH as an adjuvant, we are aware that measuring GH levels in the serum may not reflect true GH status as the hormone is released from the pituitary in a pulsatile manner, mainly during the night, and there is a diminishing output with advancing age after the second decade of life [6]. IGF-1 is generated from the liver under GH influence, and the IGF profile is known to be more stable and hence can be considered to reflect GH status [6,7] although the diagnosis of adult GH deficiency (AGHD) requires dynamic testing [6].

The data reported in this study, which examines the IGF profile against clinical parameters, shows that IGF-1, IGFBP-3 and the IGF Ratio each display a Normal distribution among the 1633 women undergoing preliminary assessment in the work-up for Assisted Reproduction. Therefore, statistical analysis for the clinical parameters examined, namely, Age, BMI and Stature, was appropriately examined by ANOVA to analyze any differences in group means.

With respect to age, we demonstrated a highly significant linear reduction in mean IGF-1 levels with advancing age across 4 age categories. For young women <35 years the mean level was 27.0 nmol/L, falling to 24.3 nmol/L for the age group 35–39 years, then 22.9 nmol/L for the age group 40–44 years and, finally, 20.1 nmol/L for those women ≥45 years. However, IGFBP-3 levels did not change significantly across the age groups remaining around 166 nmol/L. Therefore, the IGF Ratio showed a significant linear rise from 6.6 in the youngest group of women to 8.4 in the oldest group.

With respect to BMI, 8 groups were defined, categorizing from <16 kg/m^2^ to ≥40 kg/m^2^. However, none of the parameters within the IGF profile showed any significant variations across the groups. Although there was a wide standard deviation around 5 kg/m^2^, the mean levels of IGF-1 remained between 24.5 nmol/L across the groups. Similarly, there was no significant variation among the IGFBP-3 levels across the groups with mean levels around 166 nmol/L. Therefore, there was also no significant variation in the IGF Ratio according to BMI ratings, being around 7.0 across the range.

Given that stature is the most important clinical parameter to guide GH deficiency in children, we also studied the stature of the 1633 women as a specific parameter, although it comprises an essential component of the BMI estimation. Six groups were defined according to the height measurements ranging from 1.4 to 2.0 m. However, the vast majority of the subjects had heights within the range of 1.6 to 1.9 m; hence, the figures were best projected as quartiles. The mean level of IGF-1 across the height ranges was around 24.5 nmol/L without any significant variation. The inter-quartile range was 21 nmol/L to 29 nmol/L, and there was no difference in mean heights across the quartiles, being around 1.66 m. The mean level of IGFBP-3 across the height ranges was around 168 nmol/L without any significant variation. The inter-quartile range was 149 nmol/L to 186 nmol/L, and there was no difference in mean heights across the quartiles, being around 1.65 m. Consequently, there was also no significant variation in the IGF Ratio being around 7.0 across the height ranges. The inter-quartile range of IGF Ratios ranged from 5.7 to 7.8, and the mean height was around 1.66 m across the quartiles, without any significant variation.

There has been interest from the 1980s in examining IGF profiles and considering GH treatment for severe and chronic medical disorders where serum IGF-1 levels have been shown to be low [6]. A relatively recent report resurrects this idea but notes implications in interpreting low IGF-1 levels [11]. With respect to its application in assisted reproduction, there are scant reports, but one from Ankara, Turkey, in 2011 [12], examined follicular fluid levels of IGF-1 and IGFBP-3, concluding that these did not predict clinical IVF outcomes regardless of the different gonadotropin preparations. A more recent, second study from the Cornell-Weill Institute in New York [13] examined Day-2 IGF-1 serum levels in 184 women undergoing IVF and found those with levels >72 ng/mL (equivalent to 9.4 nmol/L) had a significantly higher risk of developing ovarian hyperstimulation syndrome. Such women were classified as high responders, whereas the women classified as poor responders had a significantly higher mean level of IGF-1 at 107.4 ± 60.9 ng/mL (equivalent to 14.0 ± 8.0 nmol/L). The upper level of one standard deviation recorded IGF-1 levels in this poor responder group at 165 ng/mL (equivalent to 21.6 nmol/L). Of interest, there were no significant differences in the mean IGFBP-3 levels across the 3 groups (normal, high and poor responders), but the IGF-1/IGFBP-3 ratios were significantly higher among the poor responder groups (both those pre-treated with estrogen patches or tablets and those not treated, the former group having the significantly highest ratios (16.1 ± 9.9 for normal responders, 24.0 ± 40.2 for pre-treated poor responders and 48.5 ± 46.3 for non-treated poor responders).

Of specific interest, this study from the highly reputed Cornell-Weill Institute records the IGF Ratios inverted from our own Perth study, which would provide equivalent ratios of IGFBP-3/IGF-1 at 0.09, 0.07 and 0.03. We believe the Cornell-Weill study has a concentration error (×100), and those ratios should read 9, 7 and 3, the lowest level matching our normal range (<5.0). Regardless of these differences between the Perth and New York studies, we agree that IGFBP-3 levels do not vary among the various groups presented in both studies; hence, the IGF-1 levels appear to be the most relevant for interpreting clinical parameters.

## 5. Conclusions

In conclusion, our study selected 1633 women who had their IGF profile recorded in the early follicular phase of an AC undertaken prior to any specific treatment within the PIVET assisted reproduction program. This case-series comprises complete detail on clinical parameters including age, BMI and stature. It is intended to progressively examine the IGF profile in a study on the ovarian reserve estimation with a view to evaluating the notion of AGHD underlying the poor prognosis outcomes experienced by many women undertaking assisted reproduction. In turn, this could lead to the identification of a group of women who should be offered dynamic testing and might be identified to potentially benefit from GH adjuvant treatment.

## Figures and Tables

**Table 1:** Shows the summary statistics for the IGF-1 profile for the 1633 women who completed an Assessment Cycle prior to any definitive ART treatment and from which Figure 2 is derived. The IGF-1 profile embraces serum IGF-1 levels (nmol/L) along with IGFBP-3 levels (nmol/L) and the IGF Ratio of IGFBP-3/IGF-1. The statistics include ranges, mean levels, the medians and quartiles.

Complete IGF-1 Profile—Summary Statistics			
IGF Profile	IGF-1 nmol/L	IGFBP-3 nmol/L	IGFBP-3/IGF-1 Ratio
N	1633	1633	1633
Min	8	59	2.5
Max	63	270	20.4
Sum	41,707	273,729	11,287.5
Mean	25.54	167.6	6.91
Std. error	0.17	0.73	0.04
Variance	47.67	865.72	3.20
Stand. dev	6.9	29.42	1.79
Median	25	166	6.6
25th centile	21	148.5	5.7
75th centile	29	186	7.8
Skewness	0.58	0.23	1.41
Kurtosis	390.92	334.53	747.58
Geom. mean	24.61	164.99	6.71
Coeff. var	27.03	17.55	25.89

**Table 2:** Summary statistics are shown for the 1633 women stratified according to age groups shown in Figure 3. This shows the same statistical profile depicted in Table 1, now sub-categorized for the 4 age groups with respect to IGF-1 levels, IGFBP-3 levels and the IGFBP-3/IGF-1 ratio.

	Age Groups	<35 Years	35–39 Years	40–44 Years	≥45 Years
**IGF-1 Levels vs. Age Groups—Summary Statistics**	N	929	439	220	45
	Min	10	10	8	11
	Max	63	49	52	35
	Sum	25,081	10,680	5041	905
	Mean	27.00	24.33	22.91	20.11
	Std. error	0.23	0.30	0.45	0.88
	Variance	47.44	39.02	44.29	34.74
	Std. dev	6.89	6.25	6.66	5.90
	Median	26	24	23	19
	25th centile	22	20	18	16
	75th centile	31	28	26	25
	Skewness	0.59	0.54	0.85	0.48
	Kurtosis	0.99	0.83	2.19	−0.26
	Geom. mean	26.13	23.53	21.98	19.28
	Coeff. var	25.51	25.68	29.04	29.31
	**Age Groups**	**<35 Years**	**35–39 Years**	**40–44 Years**	**≥45 Years**
**IGFBP-3 (nmol/L) vs. Age Groups—Summary Statistics**	N	929	439	220	45
	Min	59	77	59	90
	Max	265	270	270	240
	Sum	157,784	72,994	38,136	7198
	Mean	169.84	166.27	173.35	159.96
	Std. error	0.98	1.36	2.23	5.22
	Variance	900.28	812.71	1092.96	1228.00
	Std. dev	30.01	28.51	33.06	35.04
	Median	168	165	174	154
	25th centile	150	148	153	136
	75th centile	188	187	191.75	188
	Skewness	0.23	0.17	−0.01	0.31
	Kurtosis	0.56	0.11	0.56	−0.10
	Geom. mean	167.13	163.79	169.99	156.16
	Coeff. var	17.67	17.15	19.07	21.91
	**Age Groups**	**<35 Years**	**35–39 Years**	**40–44 Years**	**≥45 Years**
**c. IGFBP-3/IGF-1 Ratio vs. Age Groups—Summary Statistics**	N	929	439	220	45
	Min	2.5	3.3	3.6	4.7
	Max	13.5	16.8	20.4	17.5
	Sum	6095.3	3143.8	1670.8	377.6
	Mean	6.56	7.16	7.60	8.39
	Std. error	0.05	0.08	0.15	0.36
	Variance	2.28	3.16	5.13	5.96
	Std. dev	1.51	1.78	2.27	2.44
	Median	6.3	6.9	7.3	7.8
	25th centile	5.5	6	6.1	6.9
	75th centile	7.4	8	8.5	9.6
	Skewness	0.88	1.29	1.56	1.45
	Kurtosis	1.25	3.41	5.02	3.21
	Geom. mean	6.40	6.96	7.30	8.09
	Coeff. var	23.00	24.84	29.83	29.08

**Table 3:** Summary statistics are shown for the 1633 women stratified according to BMI groups and depicted in Figure 4. Applying the same statistical profile depicted in Table 1, now sub-categorized for the 8 BMI groups with respect to IGF-1 levels, IGFBP-3 levels and the IGFBP-3/IGF-1 ratio. Noting there are only small numbers of cases in the lowest and highest BMI groups, there are no significant differences in the mean levels, the medians or the inter-quartile ranges across the BMI spectrum.

	BMI Groups kg/m^2^	<16	16–19.9	20–23.9	24–27.9	28–31.9	32–35.9		36–39.9	≥40
**IGF-1 vs. BMI Summary Statistics**	N	93	207	352	423	258	170		76	54
	Min	17.2	16	15.8	15.2	16.2	11.8		17.1	16.6
	Max	39.5	48.7	46.5	45	46.5	43		36.8	37.1
	Sum	2318.1	5195.5	8721.2	10,455	6363.8	4303.6		1823.8	1344.2
	Mean	24.93	25.10	24.78	24.72	24.67	25.32		24.00	24.89
	Std. error	0.53	0.39	0.27	0.24	0.32	0.41		0.55	0.60
	Variance	26.21	32.09	25.34	24.41	25.65	28.96		23.09	19.26
	Std. dev	5.12	5.66	5.03	4.94	5.06	5.38		4.81	4.39
	Median	23.6	23.7	23.6	23.7	23.6	23.9		22.8	23.8
	25th centile	21.35	21.4	21	21.1	21.1	21.3		20.45	21.9
	75th centile	27.4	27.9	27.8	27.2	27.125	28.325		27	27.85
	Skewness	0.87	1.48	0.99	1.04	1.22	0.80		0.78	0.70
	Kurtosis	0.04	2.87	1.15	1.16	2.00	0.51		−0.05	0.16
	Geom. mean	24.44	24.55	24.31	24.27	24.20	24.78		23.55	24.53
	Coeff. Var	20.54	22.57	20.32	19.99	20.53	21.26		20.02	17.63
	**BMI Groups kg/m^2^**	**<16**	**16–19.9**	**20–23.9**	**24–27.9**	**28–31.9**		**32–35.9**	**36–39.9**	**≥40**
**IGFBP-3 vs. BMI Summary Statistics**	N	3	235	614	410	196		118	40	17
	Min	150	66	89	101	94		74	59	118
	Max	187	265	265	270	253		253	243	243
	Sum	516	38,984	101,786	69,417	33,358		20,086	6650	2955
	Mean	172.00	165.89	165.78	169.31	170.19		170.22	166.25	173.82
	Std. error	11.24	1.89	1.15	1.39	2.26		2.74	6.34	9.78
	Variance	379.00	842.57	810.32	792.53	1002.33		885.27	1606.81	1626.03
	Std. dev	19.47	29.03	28.47	28.15	31.66		29.75	40.09	40.32
	Median	179	163	164.5	167	169		173.5	170	166
	25th centile	150	146	147	150	149.25		151.75	147.75	141.5
	75th centile	187	184	183	187	192		188.25	196.75	207
	Skewness	−1.41	0.33	0.32	0.43	0.15		−0.29	−0.70	0.46
	Kurtosis	−2.33	0.96	0.31	0.35	−0.14		0.47	0.69	−0.80
	Geom. mean	171.24	163.31	163.33	167.00	167.19		167.41	160.37	169.53
	Coeff. var	11.32	17.50	17.17	16.63	18.60		17.48	24.11	23.20
	**BMI kg/m^2^**	**<16**	**16–19.9**	**20–23.9**	**24–27.9**	**28–31.9**		**32–35.9**	**36–39.9**	**≥40**
**IGFBP-3/IGF-1 Ratio vs. BMI Summary Statistics**	N	3	235	614	410	196		118	40	17
	Min	5.3	3.3	2.5	3.3	3.4		2.6	3.8	6.7
	Max	7.9	12.7	20.4	17.5	12.8		16.8	15.6	14.6
	Sum	19	1570.4	4114.9	2839.7	1384.1		895.3	308.1	156
	Mean	6.33	6.68	6.70	6.93	7.06		7.59	7.70	9.18
	Std. error	0.80	0.10	0.07	0.09	0.12		0.21	0.37	0.51
	Variance	1.90	2.56	2.72	3.17	2.93		5.06	5.52	4.50
	Std. dev	1.38	1.60	1.65	1.78	1.71		2.25	2.35	2.12
	Median	5.8	6.4	6.4	6.7	6.8		7.2	7.7	8.7
	25th centile	5.3	5.5	5.6	5.7	6		6.1	5.825	7.7
	75th centile	7.9	7.6	7.525	7.8	7.9		8.6	8.7	10.05
	Skewness	1.48	1.03	1.68	1.44	0.81		1.13	1.24	1.39
	Kurtosis	−2.33	1.63	8.32	4.32	0.99		2.42	2.66	1.87
	Geom. mean	6.24	6.51	6.52	6.72	6.87		7.28	7.39	8.97
	Coeff. var	21.78	23.95	24.59	25.71	24.25		29.64	30.50	23.13

**Table 4:** Summary statistics are shown for the 1633 women with IGF profiles stratified according to their stature and depicted in Figure 5. The IGF-1 levels are shown for six height categories ranging from 1.4 m to 2.0 m in as well according to their centile ranges in Table 4. Similarly, the IGFBP-3 levels are shown for the six height categories ranging from 1.4 m to 2.0 m as well as according to their centile ranges in Table 4. Finally, the IGFBP-3/IGF-1 ratios are shown for the six height categories ranging from 1.4 m to 2.0 m as well as according to their centile ranges in Table 4.

	1.4–1.49	1.5–1.59	1.6–1.69	1.7–1.79	1.8–1.89	1.9–2.0	
**IGF-1 nmol/L vs. Stature—Summary Statistics**	21	105	668	698	139	2	
	14	10	10	8	10	23	
	40	43	54	63	43	23	
	525	2530	17,015	18,092	3499	46	
	25.00	24.10	25.47	25.92	25.17	23.00	
	1.40	0.66	0.26	0.27	0.54	0.00	
	41.10	45.07	44.99	52.09	40.68	0.00	
	6.41	6.71	6.71	7.22	6.38	0.00	
	24	23	25	25	25	23	
	20.5	19	21	21	21	23	
	28.5	29	29	30	29	23	
	0.68	0.40	0.58	0.63	0.32	0.00	
	0.35	−0.31	0.97	1.12	0.28	0.00	
	24.25	23.16	24.59	24.92	24.34	23.00	
	25.64	27.86	26.33	27.85	25.34	0.00	
	**Height Quartiles (m)**	**Lowest Quartile** **IGF <21nmol/L**		**Inter-Quartile Range** **IGF 21–29 nmol/L**		**Highest Quartile** **>29 nmol/L**	
**IGF-1 nmol/L vs. Stature—Summary statistics**	N	365		869		399	
	Minimum	1.5		1.5		1.4	
	Maximum	1.8		1.9		1.8	
	Sum	600.6		1438.5		659.2	
	Mean	1.65		1.66		1.65	
	Standard error	0.00		0.00		0.00	
	Variance	0.01		0.01		0.01	
	Standard deviation	0.08		0.08		0.07	
	Median	1.6		1.7		1.7	
	25th percentile	1.6		1.6		1.6	
	75th percentile	1.7		1.7		1.7	
	Skewness	−0.03		0.10		−0.19	
	Kurtosis	−0.45		−0.24		−0.04	
	Geometric mean	1.64		1.65		1.65	
	Coefficient variation	4.82		4.63		4.41	
	**Height (m)**	**1.41.49**	**1.5–1.59**	**1.6–1.69**	**1.7–1.79**	**1.8–1.89**	**1.9–2.0**
**IGFBP-3 nmol/L vs. Stature—Summary Statistics**	N	21	105	668	698	139	2
	Min	107	103	66	59	109	173
	Max	206	244	270	265	259	221
	Sum	3216	17,146	110,246	118,544	24,206	394
	Mean	153.14	163.30	165.04	169.83	174.14	197.00
	Std. error	5.95	2.75	1.14	1.11	2.38	24.00
	Variance	742.33	793.86	872.80	863.49	789.20	1152.00
	Std. dev	27.25	28.18	29.54	29.39	28.09	33.94
	Median	150	158	163	168	173	197
	25th centile	130	143	146	150	153	173
	75th centile	179	185	184	188	190	221
	Skewness	0.12	0.46	0.17	0.23	0.47	0.00
	Kurtosis	−0.88	−0.13	0.52	0.41	0.22	−2.75
	Geom. mean	150.81	160.94	162.31	167.24	171.94	195.53
	Coeff. var	17.79	17.25	17.90	17.30	16.13	17.23
	**Height Quartiles (m)**	**Lowest Quartile** **IGFBP-3 <149 nmol/L**		**Inter-Quartile Range** **IGFBP-1 149 to 186 nmol/L**		**Highest Quartile** **IGFBP-3 >186 nmol/L**	
**IGFBP-3 nmol/L vs. Stature—Summary Statistics**	N	416		869		399	
	Min	1.5		1.4		1.5	
	Max	1.8		1.8		1.9	
	Sum	682.3		1436.35		663.2	
	Mean	1.64		1.65		1.66	
	Std. error	0.00		0.00		0.00	
	Variance	0.01		0.01		0.01	
	Std. dev	0.07		0.08		0.08	
	Median	1.6		1.7		1.7	
	25th centile	1.6		1.6		1.6	
	75th centile	1.7		1.7		1.7	
	Skewness	0.08		−0.06		0.08	
	Kurtosis	−0.27		−0.28		−0.03	
	Geom. mean	1.64		1.65		1.66	
	Coeff. var	4.53		4.65		4.53	
	**Height (m)**	**1.4–1.49**	**1.5–1.59**	**1.6–1.69**	**1.7–1.79**	**1.8–1.89**	**1.9–2.0**
**IGFBP-3/IGF-1 Ratio vs. Stature—Summary Statistics**	N	21	105	668	698	139	2
	Min	6.6	3.3	2.5	3.3	4	5.6
	Max	8.7	14.8	20.4	15.6	10.8	5.8
	Sum	22	884.5	4902.3	4525.3	942	11.4
	Mean	7.33	7.08	6.97	6.85	6.78	5.70
	Std. error	0.68	0.17	0.07	0.07	0.13	0.10
	Variance	1.40	3.73	3.70	2.80	2.20	0.02
	Std. dev	1.18	1.93	1.92	1.67	1.48	0.14
	Median	6.7	6.7	6.6	6.6	6.6	5.7
	25th centile	6.6	5.7	5.7	5.7	5.6	5.6
	75th centile	8.7	8.3	7.9	7.6	7.9	5.8
	Skewness	1.72	1.33	1.65	1.12	0.41	0.00
	Kurtosis	−2.33	3.12	5.94	2.27	−0.41	−2.75
	Geom. mean	7.27	6.84	6.74	6.66	6.62	5.70
	Coeff. var	16.15	27.28	27.59	24.43	21.90	2.48
	**Heights (m) within Ratio Ranges**	**Lowest Quartile** **Ratios 2.5 to 5.6**		**Inter-Quartile Range** **Ratios 5.7 to 7.8**		**Highest Quartile** **Ratios 7.8 to 20.4**	
**IGFBP-3/IGF-1 Ratio vs. Stature—Summary Statistics**	N	392		845		396	
	Min	1.5		1.4		1.5	
	Max	1.9		1.9		1.8	
	Sum	648.1		1398.7		651.5	
	Mean	1.65		1.66		1.65	
	Std. error	0.00		0.00		0.00	
	Variance	0.01		0.01		0.01	
	Std. dev	0.08		0.07		0.08	
	Median	1.7		1.7		1.6	
	25th centile	1.6		1.6		1.6	
	75th centile	1.7		1.7		1.7	
	Skewness	0.09		−0.08		0.08	
	Kurtosis	−0.20		−0.12		−0.44	
	Geom. mean	1.65		1.65		1.64	
	Coeff. var	4.63		4.50		4.86	

## References

[1] Yovich J., Craft I.L. (2018). Founding Pioneers of IVF: Independent innovative researchers generating livebirths within 4 years of the first birth. Reprod. Biol..

[2] Yovich J. (2020). Founding pioneers of IVF update: Innovative researchers generating livebirths by 1982. Reprod. Biol..

[3] Duncan W.C., Picton H.M., Nelson S.M., Tal R., Seifer D., Ho J., Paulson R.J., Griesinger G., Kolibianakis E., Howles C.M. (2019). How to Prepare the Egg and Embryo to Maximize IVF Success.

[4] Yovich J., Stanger J.D., Keane K.N. (2016). Cumulative Live Birth Rate: An Outmoded Term. JFIV Reprod. Med. Genet..

[5] Yovich J., Alsbjerg B., Conceicao J.L., Hinchliffe P.M., Keane K.N. (2016). PIVET rFSH dosing algorithms for individualized controlled ovarian stimulation enables optimized pregnancy productivity rates and avoidance of ovarian hyperstimulation syndrome. Drug Des. Dev. Ther..

[6] Yovich J., Regan S.L.P., Zaidi S., Keane K.N. (2019). The Concept of Growth Hormone Deficiency Affecting Clinical Prognosis in IVF. Front. Endocrinol..

[7] Stanley T.L. (2012). Diagnosis of growth hormone deficiency in childhood. Curr. Opin. Endocrinol. Diabetes Obes..

[8] Hammer Ø., Harper D.A.T., Ryan P.D. (2001). PAST: Paleontological Statistics software package for education and data analysis. Palaeontol. Electron..

[9] Ipsa E., Cruzat V.F., Kagize J.N., Yovich J.L., Keane K.N. (2019). Growth Hormone and Insulin-Like Growth Factor Action in Reproductive Tissues. Front. Endocrinol..

[10] Regan S.L.P., Knight P.G., Yovich J., Arfuso F., Dharmarajan A. (2018). Growth hormone during in vitro fertilization in older women modulates the density of receptors in granulosa cells, with improved pregnancy outcomes. Fertil. Steril..

[11] Braverman E., Oscar-Berman M., Lohmann R., Kennedy R., Kerner M., Dushaj K., Blum K. (2013). Low and Normal IGF-1 Levels in Patients with Chronic Medical Disorders(CMD) is Independent of Anterior Pituitary Hormone Deficiencies: Implications for Treating IGF-1 Abnormal Deficiencies with CMD. J. Genet. Syndr. Gene Ther..

[12] Genc G., Yılmaz N., Uygur D., Dogan M., Mollamahmutoglu L. (2011). The effect of intrafollicular IGF 1 and IGFBP 3 on IVF outcome in patients using different gonadotropins: A prospective study. J. Assist. Reprod. Genet..

[13] Man L., Lekovich J., Canon C., Rosenwaks Z., James D. (2020). Cycle day 2 insulin-like growth factor-1 serum levels as a prognostic tool to predict controlled ovarian hyperstimulation outcomes in poor responders. Fertil. Steril..

